# Spatially Heterogeneous Environmental Selection Strengthens Evolution of Reproductively Isolated Populations in a Dobzhansky–Muller System of Hybrid Incompatibility

**DOI:** 10.3389/fgene.2016.00209

**Published:** 2016-11-24

**Authors:** Samuel A. Cushman, Erin L. Landguth

**Affiliations:** ^1^USDA Forest Service, Rocky Mountain Research StationFlagstaff, AZ, USA; ^2^Division of Biological Sciences, University of MontanaMissoula, MY, USA

**Keywords:** CDPOP, computer simulations, genotype-environment associations, hybrid-incompatability, landscape genomics

## Abstract

Within-species hybrid incompatibility can arise when combinations of alleles at more than one locus have low fitness but where possession of one of those alleles has little or no fitness consequence for the carriers. Limited dispersal with small numbers of mate potentials alone can lead to the evolution of clusters of reproductively isolated genotypes despite the absence of any geographical barriers or heterogeneous selection. In this paper, we explore how adding heterogeneous natural selection on the genotypes (e.g., gene environment associations) that are involved in reproductive incompatibility affects the frequency, size and duration of evolution of reproductively isolated clusters. We conducted a simulation experiment that varied landscape heterogeneity, dispersal ability, and strength of selection in a continuously distributed population. In our simulations involving spatially heterogeneous selection, strong patterns of adjacency of mutually incompatible genotypes emerged such that these clusters were truly reproductively isolated from each other, with no reproductively compatible “bridge” individuals in the intervening landscape to allow gene flow between the clusters. This pattern was strong across levels of gene flow and strength of selection, suggesting that even relatively weak selection acting in the context of strong gene flow may produce reproductively isolated clusters that are large and persistent, enabling incipient speciation in a continuous population without geographic isolation.

## Introduction

Hybrid incompatibility refers to when hybrids between species exhibit reduced viability, lower fertility, and/or phenotypic abnormalities, and is a form of postzygotic reproductive isolation. A number of researchers have argued that hybrid incompatibility is important to the speciation process (Coyne and Orr, 2004). Dobzhansky (1937) and Muller (1942) presented models arguing that hybrid incompatibility usually evolves due to changes in at least two different genetic loci. Genetic studies strongly support the Dobzhansky–Muller model (Coyne and Orr, 2004; Seehausen et al., 2014), and a growing number of these hybrid incompatibility genes have been identified (reviewed in Johnson, 2010; Presgraves, 2010a,b).

Hybrid incompatibility can also occur between different populations of the same species (e.g., in flour beetles, Demuth and Wade, 2007; in flies, Lachance and True, 2010; in nematodes, Seidel et al., 2008, 2011). Within-species hybrid incompatibility can arise given synthetic deleterious loci, sets of loci wherein individuals with combinations of alleles at more than one locus have low fitness but where possession of one of those alleles has little or no fitness consequence for the carriers (Phillips and Johnson, 1998). Analytical studies (Phillips and Johnson, 1998; Lachance et al., 2011) showed that these synthetic alleles could reach considerably high frequencies (roughly the quartic root of the mutation rate divided by the selection coefficient) in panmictic populations under mutation-selection balance (see also, Lachance et al., 2011). Indeed, synthetic lethality and sterility has been found at appreciable frequencies in populations of *Drosophila melanogaster* (e.g., Lachance and True, 2010).

Eppstein et al. (2009) showed that limited dispersal with small numbers of mate potentials alone can lead to the evolution of clusters of reproductively isolated genotypes despite the absence of any geographical barriers or heterogeneous selection. Such clusters evolved when several loci were underdominant (heterozygotes less fit than either homozygote). Non-additive fitness effects across loci (epistasis) enhanced the likelihood of clustering. Landguth et al. (2015) extended the work of Eppstein et al. (2009) to show that underdominance is not required for clustering of reproductively isolated genotypes. Landguth et al. (2015) simulated fitness determined by epistatic interactions, in form of the well-known Dobzhansky–Muller model, and unlike past simulation studies, which consider migration of individuals between demes (e.g., Gavrilets and Vose, 2007; Gavrilets et al., 2007), they modeled genetic divergence in an individual-based framework where gene flow, genetic drift, mutation, and selection were functions of individual-based movement and spatially-explicit interactions with environment (Landguth et al., 2012).

Landguth et al. (2015) showed that hybrid incompatibility can evolve within the same population when gene flow is strongly restricted in an isolation-by-distance model. They showed that under isolation-by-distance reproductively isolated clusters could arise and persist for many generations. Most of the models of sympatric speciation wherein reproductive isolation arises in the face of moderate or strong gene flow involve the counterbalancing force of relatively strong and heterogeneous natural selection. In these models, selection enables nascent species to evolve genetic differences that are incompatible with the evolved differences in the other nascent species (Gavrilets and Vose, 2007; Gavrilets et al., 2007; Nosil and Feder, 2012). In this paper, we expand upon the Landguth et al. (2015) work and explore how adding heterogeneous natural selection on the genotypes that are involved in reproductive incompatibility affects the frequency, size and duration of evolution of reproductively isolated clusters.

## Materials and methods

### Simulation program

We used CDPOP v1.0 (Landguth et al., 2012), a landscape genetics tool for simulating the emergence of spatial genetic structure in populations resulting from specified landscape processes governing organism movement behavior. CDPOP models genetic exchange among spatially located individuals as a function of individual-based movement through mate selection and dispersal, incorporating vital dynamics (birth and death rates), and all the factors that affect the frequency of an allele in a population (mutation, gene flow, genetic drift, and selection). The landscape genetics framework of this program is such that individuals move as a probabilistic function of their environment (e.g., as habitat fragmentation increases, ability to disperse across gaps is reduced). These movement functions are scaled to a user-specified maximum dispersal and mate selection distance. This maximum movement value allows a user to control for short- and long-range movement of an organism by constraining all mate choices and dispersal distances to be within that limit, with probability specified by the user-defined movement function (e.g., inverse-square). The order of simulated events follow mate selection with given movement functions, birth and resulting Mendalian inheritance, mortality of adults, and offspring dispersal with given movement functions.

CDPOP v1.0 incorporates multi-locus selection, which is controlled via spatially-explicit fitness surfaces for each genotype under selection (Wright, 1932; Gavrilets, 2000). For example, in the case of a single two-allele locus, three relative fitness surfaces would be specified for the three genotypes (*AA, Aa*, and *aa*) from the two alleles, *A* and *a*. Selection is then implemented through differential survival of offspring as a function of the relative fitness of the offspring's genotype at the location on that surface where the dispersing individual settles (Landguth et al., 2012). CDPOP yields genetic patterns consistent with Wright–Fisher expectations when parameterized to match Wright–Fisher assumptions in simulations (Landguth and Cushman, 2010), as well as producing theoretical changes in allele frequency under selection for single and double diallelic locus (Landguth et al., 2012). For more details, see Landguth et al. (2012).

Our simulations consisted of 5000 diploid individuals with 100 biallelic loci; two of these loci were subject to selection. We initialized the 100 loci with a uniformly distributed random allele assignment (maximum allelic diversity). All loci experienced a 0.0005 mutation rate per generation (on the lower range of mammalian microsatellite rates) using the K allele model, a commonly used mutation model (Balloux, 2001; Haasl and Payseur, 2010), free recombination, and no physical linkage. Simulation parameters, other than for selection (described below), matched those in Landguth et al. (2015). Mating parameters represented a population of dioecious individuals with females and males mating with replacement. The number of offspring produced from mating was determined from a Poisson distribution (mean = 4), which produced an excess of individuals each generation to maintain a constant population size of 5000 individuals at every generation. Carrying capacity of the simulation surface was 5000 individuals. Excess individuals were discarded once all 5000 locations became occupied, which is equivalent to forcing out emigrants once all available home ranges are occupied (Balloux, 2001; Landguth and Cushman, 2010). We ran 10 Monte Carlo replicates of each simulation for 1250 generations, discarding the first 250 generations as burn-in (no selection imposed) to establish a spatial genetic pattern prior to initiating the heterogeneous landscape selection configurations.

### Simulation scenarios

**Figure 1:**
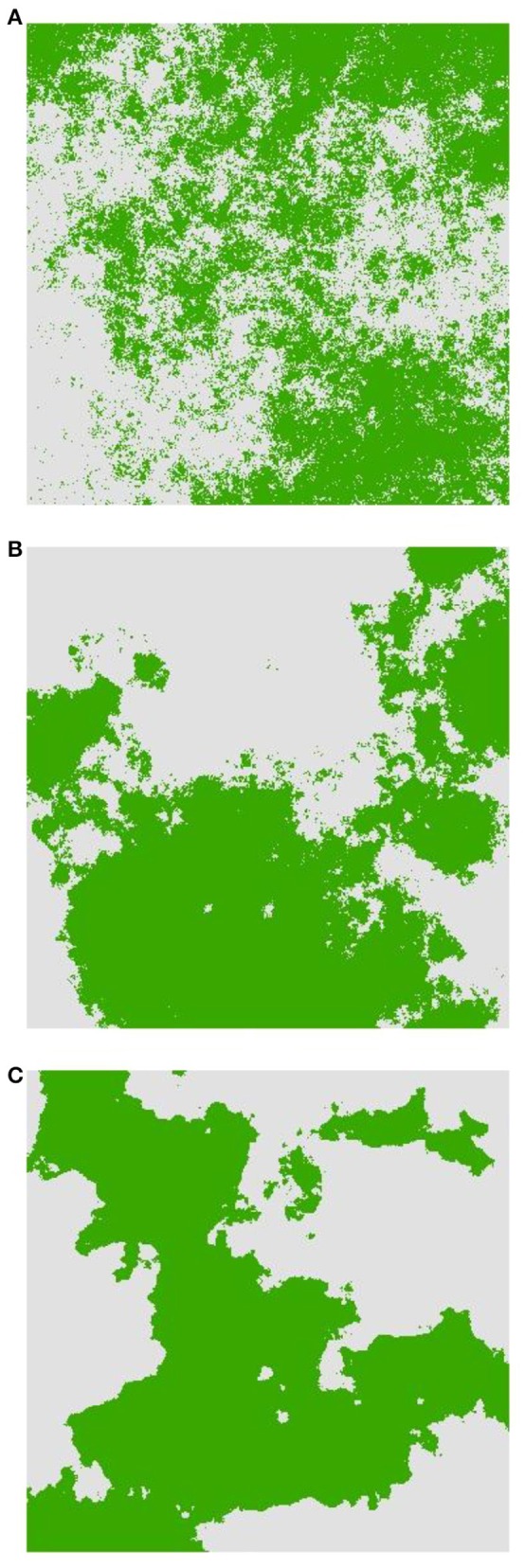
**Examples of landscape selection configurations used for simulations from least to most aggregated. (A)** H1, **(B)** H5, and **(C)** H9. Dark areas represent *AABB* habitat and light areas represent *aabb* habitat.

Our simulations combined dispersal in an isolation-by-distance (IBD) framework with heterogeneous natural selection for genotypes involved in reproductive incompatibility. The simulation modeling experiment involved all combinations of three factors (dispersal, landscape heterogeneity, and strength of selection; Figure 1).

The first factor is the degree of dispersal and we simulated six movement distances: 3, 5, 10, 15, 25, and 50% of the maximum extent of the landscape. These dispersal distances correspond to a broad range of possible dispersal destinations for a given offspring, as well as available mating partners for a given individual. Mating pairs of individuals and dispersal locations of offspring were chosen based on a random draw from the inverse-square probability function of distance, truncated with the specified maximum distance.

The second factor is the pattern of landscape heterogeneity of two habitat types providing differential selection for the genotypes involved in heterogeneous selection. Specifically, we used the neutral landscape model, QRULE (Gardner, 1999), to simulate binary landscape maps (1024 × 1024 pixels). Habitat fragmentation was controlled with the H parameter, which affects the aggregation of habitat pixels; higher values of H lead to higher levels of aggregation. The binary landscapes consisted of 50% of each of two habitat types and aggregation levels of H = 0.1 (“H1,” Figure 1A), 0.5 (“H5,” Figure 1B), and 0.9 (“H9,” Figure 1C). Heterogeneous selection acted in a discrete fashion in which different homozygous genotypes (i.e., *AABB* and *aabb*; see below) were each favored by selection in one of the two habitat types. We produced 10 replicate landscapes for each *H*-value to assess stochastic variation among simulated landscapes.

Across these different heterogeneous landscapes and dispersal distances, we tested the third factor: strength of selection, defined as the difference of relative fitness of genotypes involved in hybrid incompatibility in the two habitat types and mediated in the simulations through density-independent (i.e., environment-driven) mortality (*s*) determined by genotypes at the selected loci. Selection strengths included *s* = 0.02 or “2%,” *s* = 0.04 or “4%,” *s* = 0.08 or “8%,” *s* = 0.16 or “16%,” *s* = 0.32 or “32%,” and *s* = 0.64 or “64%” (see Table 1). Following the Dobzhansky–Muller model and the Landguth et al. (2015) simulations, we considered the two-locus (*A* and *B*), two-allele selection model (i.e., nine possible genotypes exist in the two-locus, two-allele selection model). We assumed that alleles *a* and *B* are incompatible and individuals that have these two alleles simultaneously have zero viability. This was implemented through relative fitness surfaces of 0.0 across the landscape for the genotypes *AaBB, AaBb, aaBB*, and *aaBb* as in Landguth et al. (2015). In this model, all offspring of matings between individuals *AABB* and *aabb* will have heterozygous genotype *AaBb* which will be inviable or sterile. The heterogeneous selection acting on the five remaining viable genotypes occurred relatively around *s* = 0.5 or 50% mortality. *AABB* individuals had mortality less than 50% in “*AABB*” habitat patches and experienced high mortality (>50%) if they occurred in “*aabb*” habitat patches. *AABb* individuals had mortality less than 50% but greater than the favored *AABB* individuals. Individuals with *aabb* and *Aabb* genotypes experienced the opposite selection gradient from those of *AABB* and *AABb*, respectively. For example, in the *s* = 0.02 scenarios there would be a net 2% difference in fitness between *aabb* and *AABB* genotypes in the two habitat types, with *AABB* having 51% survival in its favored habitat type, and 49% survival in its disfavored type, while *aabb* would have 51% survival in its favored type and 49% survival in its disfavored type. The *AAbb* genotypes experienced a uniform selection of *s* = 0.5 or 50% mortality across the entire surface. Table 1 lists the proportion of survival for each genotype corresponding to each relative selection strength scenario.

**Table 1:** **The proportion of survival for each genotype in ***AABB*** habitat**.

**Selection scenario (%)**	***AABB***	***AABb***	***AAbb***	***AaBB***	***AaBb***	***Aabb***	***aaBB***	***aaBb***	***aabb***
2	0.51	0.50	0.50	0.00	0.00	0.50	0.00	0.00	0.49
4	0.52	0.50	0.50	0.00	0.00	0.50	0.00	0.00	0.48
8	0.54	0.50	0.50	0.00	0.00	0.50	0.00	0.00	0.46
16	0.58	0.50	0.50	0.00	0.00	0.50	0.00	0.00	0.42
32	0.66	0.50	0.50	0.00	0.00	0.50	0.00	0.00	0.34
64	0.82	0.50	0.50	0.00	0.00	0.50	0.00	0.00	0.18

### Evaluating clusters of reproductive isolation

Following Landguth et al. (2015), we defined the occurrence of reproductive isolation in a continuously distributed population as the combination of two criteria: (1) an occurrence of a spatial cluster of individuals with genotype *AABB* that emerges simultaneously with another spatial cluster of individuals with genotype *aabb* (RI event) and (2) a RI event persisting in consecutive generations. To define an RI event, we used the density-based spatial clustering algorithm (DBSCAN; Ester et al., 1996), which finds spatial clusters if they contain sufficiently many points (*k* = 4) within a neighborhood (ε = 2000 μ; see Ester et al., 1996; Landguth et al., 2015). Then, the number of generations at which two separate clusters (*AABB* and *aabb*, respectively) emerged with the above criteria (RI events) was reported and averaged across the 10 Monte Carlo runs for each dispersal scenario. To assess persistence of RI events, we simply recorded the duration (in generations) of each RI event and reported the average time duration across each replicate and for each dispersal strategy. We also recorded the size of each RI event in terms of the number of individuals in the reproductively isolated cluster.

## Results

### Mean cluster duration

Factorial analysis of variance found highly significant main effects for landscape heterogeneity, strength of environmental selection, and dispersal ability on the mean duration that reproductively isolated clusters of individuals persisted in the simulations (Table 2). The *F*-value was more than four times higher for selection and dispersal than for landscape heterogeneity, suggesting larger differences in cluster duration across levels of selection and dispersal than levels of habitat heterogeneity. There were significant interactions between landscape heterogeneity and selection and dispersal, and weaker interaction between landscape heterogeneity and selection.

**Table 2:** **Analysis of variance table for factorial ANOVA of mean duration of reproductively isolated clusters (in generations) as function of dispersal ability (D: 3, 5, 10, 15, 25, 50% of breadth of landscape), selection (S: 2, 4, 8, 16, 32, 64% difference in relative fitness of genotypes ***AABB*** and ***aabb*** in habitat types 1 and 2 respectively), and landscape heterogeneity (Qrule H: 0.1, 0.5, 0.9) specifying the pattern of habitat types 1 and 2 in the landscape**.

**DF**	**SS**	**Mean square**	***F*-value**	**Pr > F**	**DF**
Heterogeneity	2	168,815	84,407	10.145	0.0002
Selection	5	1,832,356	366,471	44.045	2.00 × 10^−16^
Dispersal	5	2,139,692	427,938	51.433	2.00 × 10^−16^
Heterogeneity:Selection	10	282,283	28,228	3.393	0.00191
Heterogeneity:Dispersal	10	87,559	8756	1.052	0.41559
Selection:Dispersal	25	1,867,592	74,704	8.978	3.79 × 10^−11^
Residuals	50	416,016	8320		

**Figure 2:**
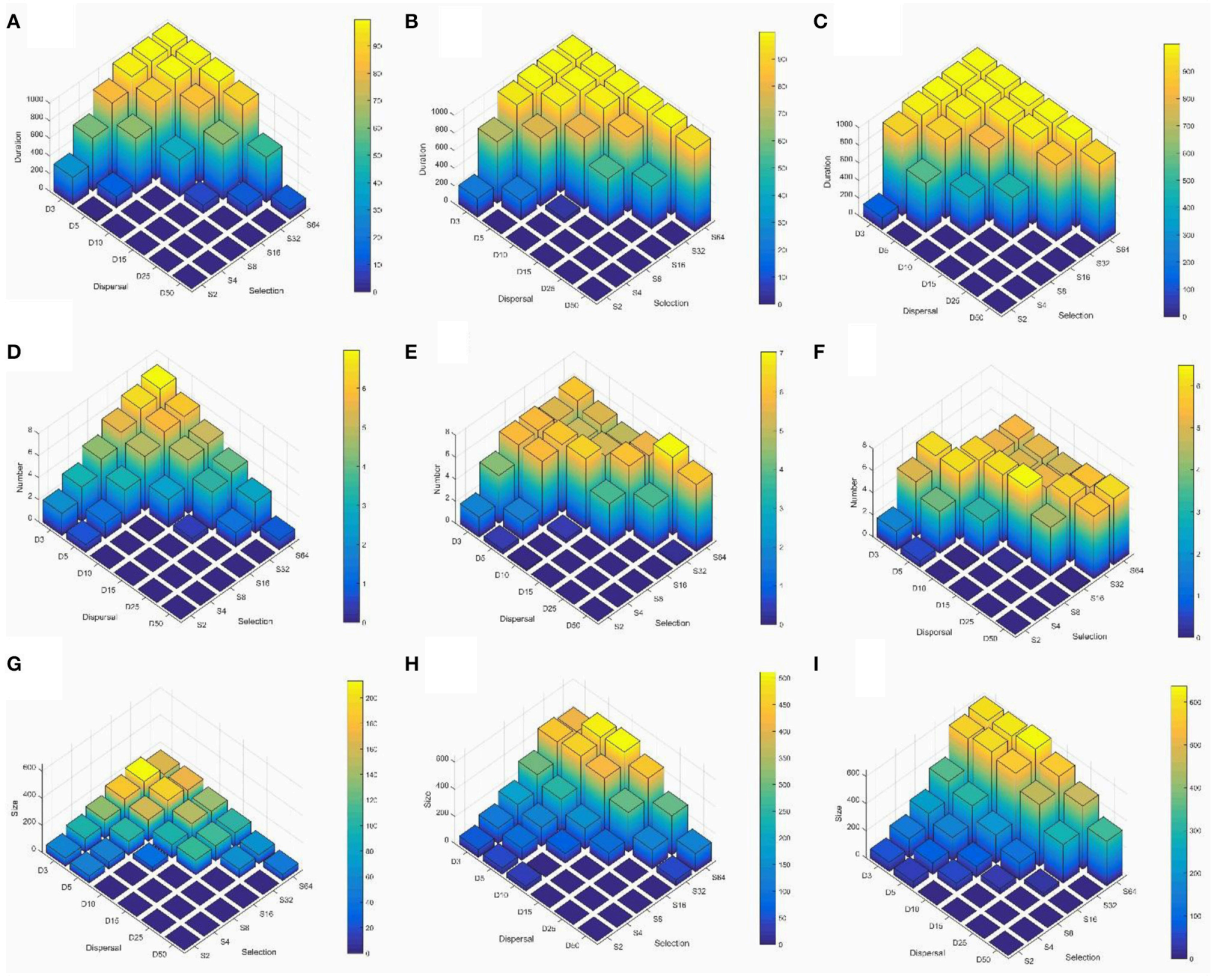
**Three-dimensional histograms of changes in the mean duration of reproductively isolated clusters of individuals (in generations; row 1, A–C)**, mean number of reproductively isolated clusters (row 2, **D–F**), and mean size of reproductively isolated clusters (individuals; row 3, **G–I**). Columns in the figure represent different levels of landscape aggregation of the two habitat types involved in environmental section of the genotypes contributing to reproductive isolation (column 1, **A,D,G** is H1, highly heterogeneous; column 2, **B,E,H** is H5, intermediate heterogeneity; column 3, **C,F,I** is H9, high aggregated patterns of the two habitat types). The 6 × 6 parameter space in each subfigure shows the combinations of six levels of dispersal (D3—3% of landscape extent, D5—5% of landscape extent, D10—10% of landscape extent, D15—15% of landscape extent, D25—25% of landscape extent, D50—50% of landscape extent) across six levels of selection (S2—2% difference in relative fitness of genotypes *aabb* and *AABB* in each of the two habitats, S4—4% difference in relative fitness, S8—8% difference in relative fitness, S16—16% difference in relative fitness, S32—32% difference in relative fitness, S64—64% difference in relative fitness). See Supplementary Videos for these histograms as they change through time.

To explore the main effects and the predominant interaction between landscape heterogeneity and dispersal we produced histograms in a dispersal × selection space, across the three levels of landscape heterogeneity (Figures 2A–C; Supplementary Video S1 duration.avi). These charts illustrate two main patterns. First, reproductively isolated clusters persist for the entire simulation time when dispersal is low and environmental selection is high. Second, the duration of reproductively isolated clusters increases across levels of dispersal and selection as landscapes become less heterogeneous. For example, at H1, the most heterogeneous configuration, reproductively isolated clusters persist for the full simulation time at combinations of dispersal between 3 and 5% and selection levels of 32 or 64 (Figure 2A; Supplementary Video S1 duration.avi). At the H5 level of heterogeneity, reproductively isolated clusters persist for the full simulation time for dispersal 3% when selection is 8 or above, at dispersal 5% when selection is 16 or above, at 10% dispersal when selection is 32 or above, and at dispersal 25% when selection is 64. The pattern continues at the highest level of aggregation, H9, when clusters have duration across the full extent of the simulation time or nearly the full extent for all combinations of dispersal and selection producing clusters (diagonal across dispersal-selection space from D3 to S64).

### Mean cluster number

Factorial analysis of variance found highly significant main effects for landscape heterogeneity, strength of environmental selection, and dispersal ability on the mean number of reproductively isolated clusters of individuals (Table 3). The *F*-value was nearly ten times higher selection and dispersal than for landscape heterogeneity, suggesting larger differences in number of isolated clusters across levels of selection and dispersal than across levels of habitat heterogeneity. There were significant interactions between landscape heterogeneity and selection and dispersal, and weaker interaction between landscape heterogeneity and selection.

**Table 3:** **Analysis of variance table for factorial ANOVA of mean number of reproductively isolated clusters as function of dispersal ability (D: 3, 5, 10, 15, 25, 50% of breadth of landscape), selection (S: 2, 4, 8, 16, 32, 64% difference in relative fitness of genotypes ***AABB*** and ***aabb*** in habitat types 1 and 2 respectively), and landscape heterogeneity (QRULE H: 0.1, 0.5, 0.9) specifying the pattern of habitat types 1 and 2 in the landscape**.

	**DF**	**SS**	**Mean square**	***F*-value**	**Pr > F**
Heterogeneity	2	35,035,209	17,517,605	9.759	0.000264
Selection	5	9.75 × 10^8^	1.95 × 10^8^	108.618	<2 × 10^−16^
Dispersal	5	6.38 × 10^8^	1.28 × 10^8^	71.09	<2 × 10^−16^
Heterogeneity:Selection	10	19,371,333	1,937,133	1.079	0.395622
Heterogeneity:Dispersal	10	6,113,877	611,388	0.341	0.965294
Selection:Dispersal	25	3.38 × 10^8^	13,537,693	7.542	8.71 × 10^−10^
Residuals	50	89,751,644	1,795,033		

The histograms (Figures 2D–F; Supplementary Video S2 number.avi) illustrate three main patterns. First, as in the case of cluster duration, the number of reproductively isolated clusters is highest when dispersal is low and environmental selection is high. Second, and contrary to cluster duration, the number of clusters shows a wave pattern moving across the dispersal × selection space toward high dispersal and low selection as the landscape becomes less heterogeneous (e.g., from H1 to H5 to H9). For example, at H1 (the most heterogeneous scenario) there is a clear peak with the largest number of reproductively isolated clusters in scenarios with the shortest dispersal (3%) and strongest selection (64), with roughly linear decay along both selection and dispersal axes (Figure 2D). However, at H5, which is an intermediate level of landscape heterogeneity, the peak of number of isolated clusters turns into a ridge running diagonally across intermediate combinations of dispersal ability and selection (Figure 2E; e.g., D3S8, D4S16, D10S16, D15S32, D25S64). The pattern continues at the highest level of landscape aggregation (lowest heterogeneity; H9) with the ridge moving diagonally toward the foreground in (Figure 2F).

### Mean cluster size

As with the other response variables (cluster duration and cluster number), factorial analysis of variance found highly significant main effects for landscape heterogeneity, strength of environmental selection, and dispersal ability on the size of reproductively isolated clusters of individuals (Table 4). The *F*-value was more twice as high for selection as for dispersal and four times higher than for landscape heterogeneity, suggesting larger differences in the size of isolated clusters across levels of selection, then dispersal, and weakest effect due to habitat heterogeneity. There were significant interactions between landscape heterogeneity and selection and dispersal, and weaker interaction between landscape heterogeneity and selection.

**Table 4:** **Analysis of variance table for factorial ANOVA of size reproductively isolated clusters (individuals) as function of dispersal ability (D: 3, 5, 10, 15, 25, 50% of breadth of landscape), selection (S: 2, 4, 8, 16, 32, 64% difference in relative fitness of genotypes ***AABB*** and ***aabb*** in habitat types 1 and 2 respectively), and landscape heterogeneity (QRULE H: 0.1, 0.5, 0.9) specifying the pattern of habitat types 1 and 2 in the landscape**.

	**DF**	**SS**	**Mean square**	***F*-value**	**Pr > F**
Heterogeneity	2	46.7	23.33	22.998	8.28 × 10^−08^
Selection	5	445.7	89.14	87.877	2.00 × 10^−16^
Dispersal	5	217.6	43.53	42.91	2.00 × 10^−16^
Heterogeneity:Selection	10	20.7	2.07	2.036	0.0489
Heterogeneity:Dispersal	10	2.8	0.28	0.272	0.9846
Selectoin:Dispersal	25	135.3	5.41	5.336	2.53 × 10^−07^
Residuals	50	50.7	1.01		

The histograms displaying size of reproductively isolated clusters across combinations of dispersal ability and strength of environmental selection (Figures 2G–I; Supplementary Video S3 size.avi) show a pattern similar to those for cluster duration, except that in the case of cluster size selection seems to have a substantially larger effect than dispersal ability. Specifically, at all levels of habitat heterogeneity (H) the size of clusters of reproductively isolated individuals is highest at when selection is strong and dispersal is limited, but large clusters can persist at high levels of selection even when dispersal is relatively broad-scale (e.g., S32–S64 when D10–D15), while the converse is not true; clusters remain small when selection is weak even when dispersal is limited (e.g., S2–S8 when D3–D10). Second, there is a large effect of changing patterns of heterogeneity of the landscape features driving environmental selection of the genotypes involved in reproductive isolation (Figures 2E–G; Supplementary Video S3 size.avi). For example, when H is 1 (highest level of heterogeneity) the largest cluster sizes are around 220 individuals (at D3S32). At H5 (intermediate heterogeneity) clusters of this size are found at levels of D3–D25 × S32–S64, and the largest cluster sizes exceed 450 individuals at combinations of dispersal and selection D3–D5 × S32–S64, and the largest clusters of over 500 individuals emerge at dispersal levels of between D5–D10 and selection level S64. The pattern continues at H9 (highest habitat aggregation) where clusters of over 630 reproductively isolated individuals emerge and clusters larger than 500 individuals are found at combinations of dispersal D3–D15 across selection levels of S32–S64 (Figure 2G).

## Discussion

Landguth et al. (2015) found that short-range dispersal strategies lead to the evolution of clusters of reproductively isolated genotypes despite the absence of any geographic barriers or heterogeneous selection. In addition, they found that clusters of genotypes that are reproductively isolated from other clusters can persist when migration distances are restricted such that the number of mating partners is below about 350 individuals. From these results they argued that under strong selection clusters of incompatible genotypes will readily evolve within continuously distributed populations when dispersal distances and potential mating choices are small relative to entire landscape extents and population size, respectively. Short mating distances reduce the rate at which genes moved through the population and reduce local effective population sizes such that local genetic structure would be maintained and not swamped by the homogenizing effects of high rates of gene flow. When mating and dispersal are very limited, reproductive isolation frequently evolves and reproductively isolated clusters may be highly persistent over time.

In this paper, we show that adding heterogeneous selection for the genotypes involved in reproductive isolation led to dramatic increases in the duration, number, and size of reproductively isolated patches. Landguth et al. (2015) found that reproductively isolated clusters do not evolve when dispersal is >10% of the extent of the population, and that few clusters evolve and these only persist a short time when dispersal is >5% of the extent of the population. In strong contrast, we found that when there is spatially heterogeneous selection on genotypes involved in reproductive isolation, reproductively isolated clusters can evolve even at very high levels of dispersal, and these clusters can achieve very large size and very long duration, with number, size and duration increasing with the strength of selection.

We also found that strength of selection and dispersal ability affect the size, duration, and number of isolated clusters in roughly the same degree, and much more so than does the heterogeneity of the landscape. However, landscape heterogeneity does have substantial effects, such that when there is extremely high heterogeneity reproductively isolated clusters are less likely to emerge since there is a highly mixed pattern of selection that inhibits formation of large, aggregated clusters. This suggests that in evolutionary landscape genetics, as well as neutral differentiation (e.g., Cushman et al., 2012, 2013), there may be threshold effects where landscape fragmentation limits emergence of reproductively isolated clusters. However, in contrast to the effect of habitat fragmentation on emergence of neutral genetic structure, in which genetic differentiation only occurs at high levels of landscape heterogeneity, evolution of reproductive isolation is facilitated by highly blocky landscapes with relatively low fragmentation.

**Figure 3:**
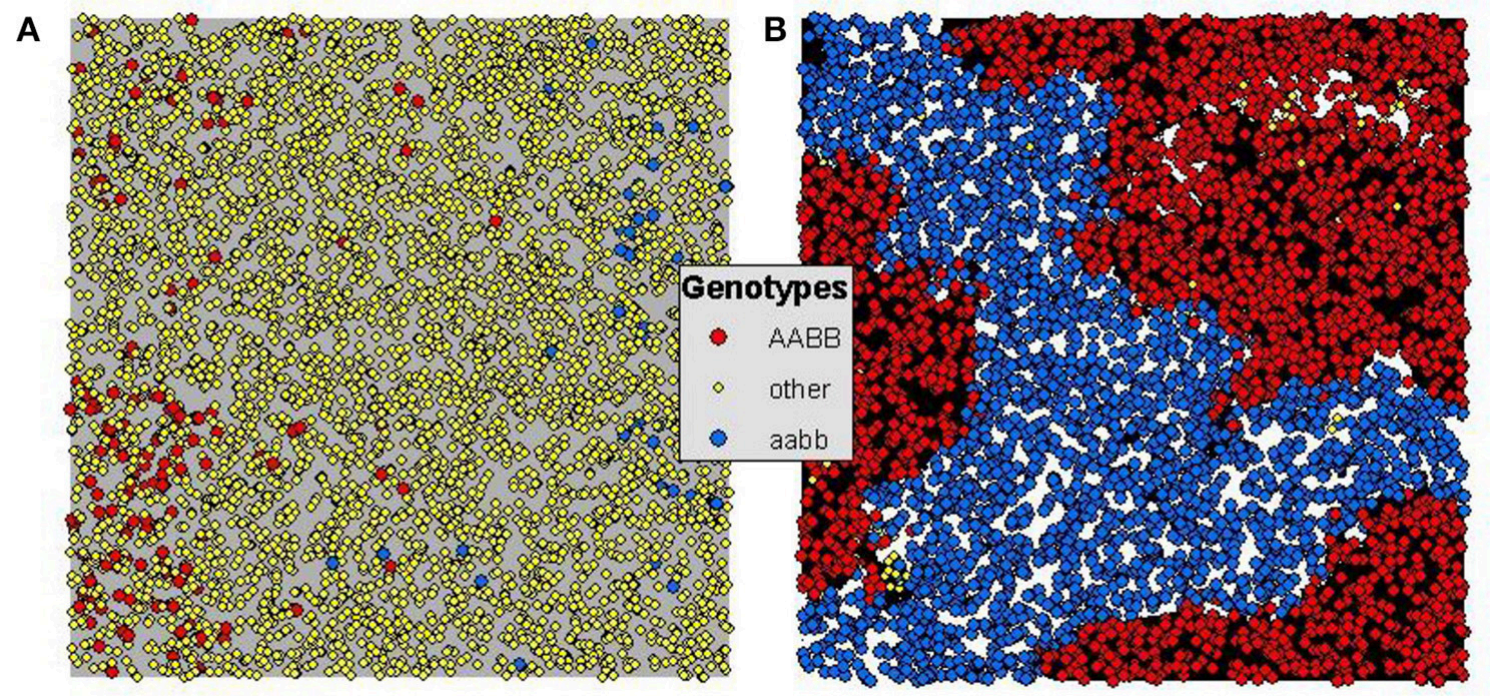
**Generation 1250 for 5% maximum dispersal scenarios of (A)** uniform selection (i.e., Landguth et al., 2015) and **(B)** heterogeneous selection of *H* = 0.9 and *S* = 64. Orange dots indicate genotype *AABB*, yellow dots indicate genotype *aabb*, and all other genotypes as green dots. **(A)** Shows the pattern of genotypes (red and blue mutually reproductively isolated and yellow compatible with both) in the pure isolation-by-distance framework of Landguth et al. (2015) without heterogeneous selection. **(B)** Shows the pattern of genotypes for a heterogeneous selection scenario with dispersal limited to 5% of the extent of the population and selection set at 64. In **(A)** there are few and small reproductively isolated clusters and these are not truly isolated as the yellow genotypes provide a genetic bridge for gene flow between red and blue. In contrast in **(B)** there is nearly complete elimination of the yellow “bridge” genotypes, and extensive, large and immediately adjacent patches of mutually isolated genotypes (red next to blue).

In addition to the much larger total number, size, and duration of reproductively isolated patches when there is environmental selection, the pattern of cluster adjacency changes in critical ways that enable persistence of reproductively isolated clusters and therefore the potential for incipient speciation. Specifically in the Landguth et al. (2015) simulation, reproductively isolated clusters evolved only as a function of reproductive isolation and gene flow restriction by isolation-by-distance. This resulted in patterns of clusters in the landscape where putatively “reproductively isolated” clusters were rarely adjacent to clusters of individuals that were actually incompatible with them (Figure 3). They were most often adjacent to individuals that were not reproductively isolated from them, and clusters that were reproductively incompatible with them typically existed in other parts of the landscape with non-incompatible individuals in between. These non-incompatible individuals form a genetic “bridge” allowing gene flow between the putatively isolated clusters. While based on the criteria used by Landguth et al. (2015) this qualifies as evolution of reproductively isolated clusters, these clusters they were not isolated in the sense that individuals in these clusters could breed with the individuals that were adjacent to them, and could transfer genes between “isolated” clusters through the “bridge” of these compatible intervening individuals (Figure 3).

In contrast, when we added environmental selection on the genotypes involved in hybrid incompatibility very strong patterns of adjacency of mutually incompatible genotypes emerged such that these clusters were truly reproductively isolated from each other as there were no other reproductively compatible “bridge” individuals in the intervening landscape to allow gene flow between the clusters. This pattern was very strong across levels of gene flow and strength of selection, suggesting that even relatively weak selection acting in the context of strong gene flow may produce reproductively isolated clusters that are large and persistent, enabling incipient speciation in a continuous population without geographic isolation.

There are several lines of future work which should be explored to extend the scope of what was found in this paper. First, this paper used a simple two-locus model of hybrid incompatibility. While this is a model that is widely used in theoretical evolutionary ecology (Dobzhansky, 1937; Muller, 1942; Coyne and Orr, 2004) and applies to some real-world populations (Demuth and Wade, 2007, in flies, Lachance and True, 2010; in nematodes, Seidel et al., 2008, 2011), the majority of microevolutionary processes are likely mediated through polygenic selection in which many loci each contribute relatively small fitness effects. This paper serves as an initial analysis of a simple, classical model of two locus selection which provides clear theoretical insight. However, future work should explore how landscape heterogeneity, strength of selection, and dispersal ability interact within the context of multiple loci/allele selection (e.g., de Villemereuil et al., 2014) and how these factors influence the detection of local adaptation (e.g., genotype-environment associations; Bierne et al., 2011; Forester et al., 2016). In addition, future work should explore how underdominance, epistasis, and synonymous vs. nonsynonymous mutations interact in their influence on evolution of reproductively isolated clusters in continuous populations in heterogeneous landscapes. In addition, it will be important to combine simulation experiments with empirical studies and experiments (e.g., Cushman, 2014) to develop robust understanding of how landscape heterogeneity, patterns of gene flow and selection, and dispersal ability affect population differentiation and evolution. Simulation experiments such as presented here can describe the processes affecting populations and identify the conditions under which they have important influences. However, models without data are not compelling (Cushman, 2014). It is essential to confront these models with empirical data on the actual patterns of genetic differentiation in complex landscapes, and to confirm the fitness relationships underlying these patterns in experimental studies such as common gardens (Cushman, 2014). Thus, we suggest future research that will combine simulation, experimentation, and large-scale population-wide empirical modeling of the influences of landscape heterogeneity, gene flow and strength of selection on the emergence of reproductive isolation.

## Author contributions

SC and EL designed research. EL ran simulations. SC and EL performed the analyses, interpreted the data, and wrote the manuscript.

### Conflict of interest statement

The authors declare that the research was conducted in the absence of any commercial or financial relationships that could be construed as a potential conflict of interest.

## Supplementary material

Supplementary Videos **AVIs of three-dimension histograms through time for (S1) change in mean duration of reproductively isolated clusters **(S2)**, mean number of reproductively isolated clusters, and **(S3)** mean size of reproductively isolated clusters**.
